# Comparative Analysis of Artificial Intelligence (AI) Languages in Predicting Sequential Organ Failure Assessment (SOFA) Scores

**DOI:** 10.7759/cureus.59662

**Published:** 2024-05-05

**Authors:** Fuat H Saner, Yasemin M Saner, Ehab Abufarhaneh, Dieter C Broering, Dimitri A Raptis

**Affiliations:** 1 Organ Transplant Center of Excellence, King Faisal Specialist Hospital and Research Centre, Riyadh, SAU; 2 Department of Urology, Medical Center University Duisburg-Essen, Essen, DEU

**Keywords:** chatgpt, large language models, artificial intelligence, perplexity, bard, sofa score

## Abstract

Purpose: The Sequential Organ Failure Assessment (SOFA) score plays a crucial role in intensive care units (ICUs) by providing a reliable measure of a patient's organ function or extent of failure. However, the precise assessment is time-consuming, and daily assessment in clinical practice in the ICU can be challenging.

Methods: Realistic scenarios in an ICU setting were created, and the data mining precision of ChatGPT 4.0 Plus, Bard, and Perplexity AI were assessed using Spearman's as well as the intraclass correlation coefficients regarding the accuracy in determining the SOFA score.

Results: The strongest correlation was observed between the actual SOFA score and the score calculated by ChatGPT 4.0 Plus (*r *correlation coefficient 0.92) (p<0.001). In contrast, the correlation between the actual SOFA and that calculated by Bard was moderate (*r*=0.59, p=0.070), while the correlation with Perplexity AI was substantial, at 0.89, with a p<0.001. The interclass correlation coefficient analysis of SOFA with those of ChatGPT 4.0 Plus, Bard, and Perplexity AI was ICC=0.94.

Conclusion: Artificial intelligence (AI) tools, particularly ChatGPT 4.0 Plus, show significant promise in assisting with automated SOFA score calculations via AI data mining in ICU settings. They offer a pathway to reduce the manual workload and increase the efficiency of continuous patient monitoring and assessment. However, further development and validation are necessary to ensure accuracy and reliability in a critical care environment.

## Introduction

The Sequential Organ Failure Assessment (SOFA) score is used to evaluate the performance of various organ systems in critically ill patients, including neurological function, coagulation, liver, kidney, respiration, and hemodynamics. Each category is assessed and assigned a score based on the observed patient data in this scoring system. Initially developed for categorizing critically ill patient groups, such as those suffering from sepsis or acute respiratory distress syndrome (ARDS), the SOFA score aims to predict patient outcomes by analyzing organ function. This assessment involves six key criteria that represent different organ systems: respiratory, cardiovascular, renal, neurological, hepatic, and hematological. A score ranging from 0 to 4 for each system is given, where 0 represents normal functioning and 4 indicates severe impairment. This systematic approach helps understand the extent of organ failure and may guide medical intervention in critical care settings [1,2].

Even though the SOFA score was introduced in 1996 [2], the application rate still needs to improve in numerous intensive care units (ICUs) [3]. This can be attributed to several factors. The comprehensive nature of the SOFA score involves meticulous effort, as it requires the integration of various laboratory values and a detailed clinical evaluation of the central nervous system. These processes are inherently time-intensive. Consequently, in such a context, the role of artificial intelligence (AI) languages becomes increasingly significant. These AI systems hold the potential to streamline and expedite these complex assessments, thereby enhancing the efficiency and effectiveness of patient care in these critical settings [4].

AI, mainly equipped with natural language processing (NLP) technology, can compile and articulate comprehensive medical histories and accurately gather essential scores vital for the comparative analysis of different patient groups. This process is also known as data mining. This technological advancement is particularly beneficial in the setting of intensive care medicine. In this field, complex treatment protocols necessitate meticulous documentation and assessment. Notably, the SOFA score has recently become a requisite for billing and reimbursement procedures with health insurance entities. Integrating AI in healthcare may simplify the administrative aspects and ensure more precise and efficient handling of critical patient data, a key in the management of intensive care treatments.

The aim of this study was to assess the data mining capability and precision of three commonly used and available AI large language models in determining the SOFA score of patients in the ICU setting.

## Materials and methods

ChatGPT, developed by OpenAI in San Francisco, California, United States (OpenAI. 2023. "ChatGPT, version 4." Accessed April 19. https://chat.openai.com/), and Bard (Bard (2024). Google AI. Retrieved from https://bard.google.com) and Perplexity (Perplexity AI. (2023). Perplexity (AI-powered search engine). https://www.perplexity.ai/​) were utilized to evaluate SOFA. All clinical scenarios presented were fictitious and did not represent real patient data; hence, ethical approval was not necessitated. Initially, 10 critical care scenarios were developed, with a SOFA score meticulously calculated for each. The simulated patient data from these scenarios were presented to three common AI tools: ChatGPT 4.0 Plus, Bard, and Perplexity AI. The assessment process involved a detailed evaluation of each organ system by these tools. Subsequently, the individual organ system assessments and the final SOFA scores derived from these evaluations were systematically recorded in a database. This file was specifically prepared for conducting further in-depth statistical analysis and evaluation. The detailed narratives of these fictional cases are available in the Appendices accompanying this study.

Statistical analysis was performed using R version 3.3.2 (R Core Team, GNU GPL v2 License) and R Studio version 1.0.44 (RStudio, Inc. GNU Affero General Public License v3, Boston, Massachusetts, United States, 2016) with the graphical user interface (GUI) rBiostatistics.com alpha version (rBiostatistics.com, London, United Kingdom, 2017). Data are presented as the median and the 25th to 75th percentiles for samples with non-normal distribution and as mean±standard deviation (SD) for normally distributed samples. The Spearman rank correlation was performed to analyze the relationship between calculated SOFA, ChatGPT 4.0 Plus, Bard, and Perplexity AI. Additionally, the intraclass correlation coefficient (ICC) analysis was conducted to assess the overall correlation between the different AI tool calculations and the actual SOFA score. 

## Results

The comparative analysis of SOFA scores across different organ systems is provided in Table 1, as assessed by calculated methods and AI tools (ChatGPT 4.0 Plus, Bard, and Perplexity AI). It presents median (IQR) values for each system: central nervous system (CNS), cardiovascular system (CVS), respiration, renal, liver, and coagulation. The overall SOFA scores are also compared as listed in Table 1.

**Table 1:** Spearman rank correlation of calculated SOFA score with SOFA assessment of ChatGPT 4.0 Plus SOFA, Bard SOFA, and Perplexity AI SOFA SOFA: Sequential Organ Failure Assessment

	Calculated SOFA	ChatGPT 4.0 Plus SOFA	Bard SOFA	Perplexity AI SOFA	P-value
Calculated SOFA	1.00	0.96	0.69	0.90	
ChatGPT 4.0 Plus SOFA	0.96	1.00	0.76	0.88	<0.001
Bard SOFA	0.69	0.76	1.00	0.65	0.07
Perplexity AI SOFA	0.90	0.88	0.66	1.00	<0.001

**Figure 1:**
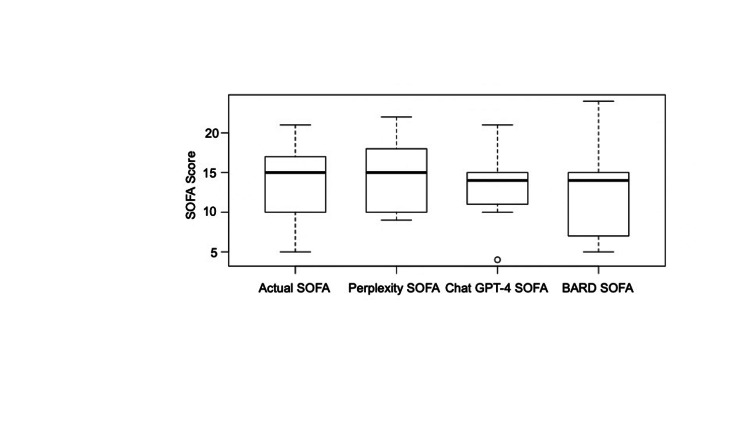
Comparing calculated SOFA score with Perplexity AI, ChatGPT 4.0 Plus, and Bard scores SOFA: Sequential Organ Failure Assessment

The Spearman rank correlation of the SOFA score with calculations performed by ChatGPT 4.0 Plus, Bard, and Perplexity AI are presented in Table 2. This shows the correlation coefficients between each pair of assessments. The highest correlation was observed with ChatGPT 4.0 Plus (r=0.96; p<0.001) followed by Perplexity AI (r=0.90; p<0.001) and Bard (r=0.69; p=0.07) (Figure 1).

**Table 2:** Correlation of calculated SOFA predicted with ChatGPT 4.0 Plus, Bard, and Perplexity AI Data are given as median and 25th and 75th quartiles, as they are not normally distributed CNS: central nervous system; CVS: cardiovascular system; SOFA: Sequential Organ Failure Assessment

	Calculated	ChatGPT 4.0 Plus	Bard	Perplexity AI
CNS	3.00 (2.25; 3.00)	3.00 (2.25; 3.00)	4.50 (3.00; 8.00)	3.00 (1.50; 4.00)
CVS	3.50 (3.00; 4.00)	2.50 (2.00; 3.00)	1.00 (0.00; 2.00)	3.50 (3.00; 4.00)
Respiration	1.00 (1.00; 2.75)	2.00 (2.00; 3.00)	2.00 (1.00; 4.00)	2.50 (1.00; 3.00)
Renal	2.00 (2.00; 3.00)	2.00 (2.00; 3.00)	1.50 (1.00; 2.00)	3.00 (2.00; 3.00)
Liver	2.00 (1.00; 2.00)	2.00 (1.00; 2.00)	0.50 (0.00; 1.00)	1.50 (1.00; 2.00)
Coagulation	2.00 (1.00; 2.75)	1.50 (1.00; 2.75)	0.00 (0.00; 1.00)	2.00 (1.00; 3.00)
SOFA	15.00 (10.25; 16.25)	14.00 (11.00; 15.00)	14.00 (8.00; 14.75)	15.00 (10.5; 18.00)

**Figure 2:**
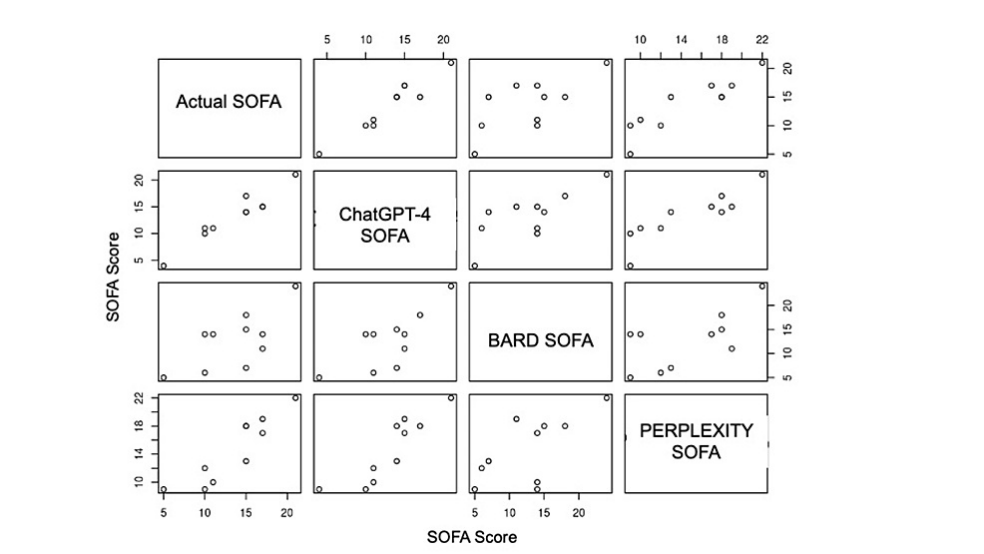
ICC between calculated SOFA score, ChatGPT 4.0 Plus score, Bard score, and Perplexity AI score ICC: interclass correlation coefficient; SOFA: Sequential Organ Failure Assessment

The ICC values with 0.94 indicate good levels of agreement (Table 3 and Figure 2).

**Table 3:** ICCs ICC: interclass correlation coefficients; F: F-statistics; df1: degree of freedom 1; df2: degree of freedom 2

	Type ICC	F	df1	df2	P-value	Lower bound	Upper bound
Single raters absolute	ICC1 0.78	15	9	30	<0.001	0.55	0.93
Single random raters	ICC2 0.78	16	9	27	<0.001	0.55	0.93
Single fixed raters	ICC3 0.79	16	9	27	<0.001	0.56	0.93
Average raters absolute	ICC1k 0.93	15	9	30	<0.001	0.83	0.98
Average random raters	ICC2k 0.93 1	16	9	27	<0.001	0.83	0.98
Average fixed raters	ICC3k 0.94 1	16	9	27	<0.001	0.83	0.98

## Discussion

The strong correlation between the actual SOFA and that data minded by ChatGPT 4.0 Plus indicates the potential utility of AI models in such medical assessments. However, such models will need to be further improved to ensure high accuracy. 

The relatively weaker correlation (r=0.69) with the "Bard" is particularly notable. An r-value of 0.69, while indicating some correlation, does suggest a level of randomness or inconsistency in Bard's assessment of SOFA scores compared to the calculated scores. This could imply that the Bard method might need to be revised to accurately assess the severity of organ failure in patients, at least in comparison to the calculated SOFA scores or the assessments made by ChatGPT 4.0 Plus.

These findings could have implications for integrating AI in medical diagnostics and patient monitoring, highlighting areas where AI can be effectively utilized and where further refinement is needed. There are many scoring systems for ICU patients. However, the SOFA score is widely used in ICUs to track a patient's status and determine the extent of organ function or rate of failure. The SOFA score was validated in various studies and is considered helpful in predicting the clinical outcomes of critically ill patients. Moreover, the SOFA score's prognostic accuracy has been validated in different patient populations, including those undergoing cardiac, thoracic, and vascular surgery [5]. The utility of the SOFA score extends beyond just assessing organs. Additionally, the SOFA score is a familiar tool to critical care physicians and provides a standardized, numeric score that can be used to compare patient status [3].

Despite AI language models not yet being a substitute for manual SOFA calculations, the high accuracy of ChatGPT 4.0 Plus and Perplexity AI about manually calculated SOFA scores is noteworthy. This finding underscores the continuous advancement of AI tools for enhancing clinical settings.

Zúñiga Salazar et al. [6] evaluated the use of AI chatbots, like ChatGPT, Google Bard, and Microsoft Bing AI, for the effectiveness of these chatbots in distinguishing between medical emergencies and non-emergency situations.

The results revealed that AI chatbots generally identified more cases as emergencies (about 12-15% more) than human reviewers. They were less likely to classify cases as non-emergencies, 35% less often than the reviewers. This is in line with our findings. While ChatGPT 4.0 Plus shows the highest correlation with the SOFA score calculated by humans, Perplexity AI and, mainly, Bard could have been more accurate.

Iannantuono et al. [7] reported on using large language models in oncology, particularly assessing their applications in cancer care. The authors evaluate the performance of ChatGPT in comparison with other large language models like Google Bard, Chatsonic, and Perplexity. While ChatGPT has shown proficiency in providing information on cancer screening and management, it has also demonstrated a significant error rate and a tendency to provide outdated information. Similar findings were also seen in our study, underscoring the importance of expert-driven proof of AI-made chats to prevent AI-associated hallucination.

In another study, Patil et al. [8] aimed to compare the performance of ChatGPT 4.0 and Google's Bard in answering radiology board examination questions. The results showed that ChatGPT 4.0 was significantly more accurate than Bard (87.11% vs 70.44%), but ChatGPT took longer to respond (26.79 seconds) than Bard (7.55 seconds) per question. The results of this study highlighted ChatGPT's superior knowledge compared to Bard's. However, like in our study, both chatbots have limitations, often providing incorrect or illogical answers and only sometimes addressing the educational content of the question. 

Another interesting study [9] evaluated the three AI models, ChatGPT, Bard, and Bing AI, examining their applications in financial analysis and task automation, completely different from medicine. Of note, although this is an entirely different field, the authors came up with similar results to those in the medical field. The conclusion stresses that these AI languages hold significant promise for the finance industry.

Our study has some strengths and weaknesses. We created fictive scenarios to simulate an ICU environment for evaluating the SOFA score's precision using AI models like ChatGPT 4.0 Plus, Bard, and Perplexity AI. We then compare them with the calculated SOFA score. The conclusion drawn from this study underscores the significant potential of AI tools like ChatGPT 4.0 Plus to assist with complex medical assessments in ICUs. They offer a valuable avenue to reduce manual effort and enhance the efficiency of patient monitoring. However, the study also highlights the need for further development and rigorous validation of these tools, particularly for those like Bard, to ensure their accuracy and reliability in high-risk environments such as critical care. This study is a promising step towards integrating AI into healthcare, though it also cautions against the premature application of these technologies without thorough vetting and improvement.

All these studies, including our study, examined the efficacy of AI chatbots, such as ChatGPT 4.0 Plus, in various domains including healthcare and finance. All studies showed good accuracy, particularly in ChatGPT 4.0 Plus, less in Bard or Perplexity AI. However, the assessment should be precise to avoid making the wrong medical decision, particularly when it comes to scoring systems, which predict patient outcomes. The same issue is for the financial sector; here, accuracy is also good but still needs improvement.

However, it is crucial to critically analyze and address the limitations and ethical concerns associated with these technologies. 

Ethical and privacy concerns could be a concern for AI systems. AI systems, including language models, process vast amounts of personal and sensitive data. There are significant concerns regarding the privacy and security of patient data, as AI systems can be vulnerable to data breaches and unauthorized access. Ethical dilemmas also arise from the potential misuse of AI, such as discrimination and bias in AI algorithms, which can lead to unequal treatment outcomes. 

Bias and fairness should be critically assessed. AI models are only as good as the data they are trained on. If the training data is biased, the AI's decisions will likely reflect these biases. This can lead to disparities in healthcare delivery, where certain demographic groups may receive less accurate diagnoses or suboptimal treatment recommendations.

Transparency of many AI systems, particularly those based on deep learning, are often referred to as "black boxes" because their decision-making processes are not easily understandable by humans. This can lead to a loss of trust between the physician and the patient.

Dependence and de-skilling are major issues. More reliance on AI tools can lead to de-skilling healthcare professionals, as they may become overly dependent on automated systems for diagnosis and treatment decisions [10] compared to a pilot who can only take off and land with electronic assistance and can no longer fly an aircraft manually.

The implementation of AI languages in daily praxis can cause many issues. The integration of AI into existing healthcare systems poses significant challenges, including technical integration with existing electronic health records, alignment with clinical workflows, and the need for substantial training for healthcare providers [10]. These challenges can lead to increased costs and may slow down the adoption of AI technologies.

As with any study, there are limitations. The reliance on AI algorithms, including ChatGPT 4.0 Plus, may not capture the full clinical context or nuances of a patient's condition as observed by experienced healthcare professionals. Another area for improvement is the potential variability in data input quality, as inaccuracies in the initial data provided to the AI models could lead to erroneous SOFA score calculations. Finally, the study's findings are based on simulations within a controlled environment and may only partially replicate the dynamic and complex nature of real-world ICU settings.

One significant strength of the study is its innovative approach to using advanced AI technologies like ChatGPT 4.0 Plus for enhancing the precision and efficiency of critical care assessments, demonstrating a high correlation with actual SOFA scores. Another strength lies in the use of rigorous statistical analysis, including Spearman's and ICCs, to validate the reliability and accuracy of AI-generated SOFA scores against standard clinical evaluations. Lastly, the study highlights the potential of AI tools to significantly reduce the workload of healthcare professionals in ICUs by automating routine but critical tasks.

Our study focused on simulating an ICU environment to assess the precision of AI models like ChatGPT 4.0 Plus, Bard, and Perplexity AI in evaluating the SOFA score. It highlighted the potential of AI tools in assisting with complex medical assessments, particularly in ICUs, by reducing manual effort and enhancing patient monitoring efficiency. However, the study also underscored the necessity for further development and rigorous validation of these AI tools, especially in critical care settings, to ensure their accuracy and reliability.

## Conclusions

This study highlights the potential benefits of AI tools like ChatGPT 4.0 Plus, Bard, and Perplexity AI in helping healthcare professionals quickly and accurately assess organ dysfunction using SOFA scores in critical care. While the results show promise in terms of efficiency and automation, the study emphasizes the need for further research and careful validation before these AI tools can be widely used in clinical settings. It also suggests that the best approach may involve combining AI with human expertise to enhance patient care, with AI serving as a support tool rather than a replacement for clinical judgment. The strong ICCs and correlations reported suggest a solid basis for the future exploration of AI in critical care, indicating a promising path for improving patient outcomes with technology.

## Appendices

**Table 4:** Patient 1 MAP: mean arterial pressure

Patient 1	
Neurological	Open eyes to pain. Inappropriate words. Flexion to pain
Cardiovascular	MAP above 65 on norepinephrine 0.03 µg/kg/min. Lactate 5.3
Respiratory	Intubated. FiO_2_=100%. paO2=75 mmHg
Renal	Creatinine: 2.5 mg/dl
Gastrointestinal	Bilirubin: 5 mg/dl
Hematology	Platelets: 99/nl

**Table 5:** Patient 2 MAP: mean arterial pressure

Patient 2	
Neurological	Open eyes to pain. Inappropriate words. Flexion to pain
Cardiovascular	MAP above 65 on norepinephrine 0.03 µg/kg/min
Respiratory	Intubated. FiO2=30%. paO2=75 mmHg
Renal	Is on dialysis. Urine output <200 ml/day
Gastrointestinal	Bilirubin: 5 mg/dl
Hematology	Platelets: 30/nl

**Table 6:** Patient 3 MAP: mean arterial pressure

Patient 3	
Neurological	Open eyes spontaneously. Alert/oriented. Obeys command
Cardiovascular	MAP above 65 on norepinephrine 0.1 µg/kg/min
Respiratory	Mechanical ventilation. FiO2=50%. paO2=75 mmHg
Renal	Acute kidney injury II. Urine output: 400 ml
Gastrointestinal	Bilirubin: 2 mg/dl
Hematology	Platelets: 75/nl

**Table 7:** Patient 4 MAP: mean arterial pressure

Patient: 4	
Neurological	No eye opening. Incomprehensible sounds. No motor response
Cardiovascular	MAP above 65 on norepinephrine 0.5 µg/kg/min
Respiratory	Mechanical ventilation. FiO2=50%. paO2=150 mmHg
Renal	Urine output: 400 ml
Gastrointestinal	Bilirubin: 12 mg/dl
Hematology	Platelets: 120/nl

**Table 8:** Patient 5 MAP: mean arterial pressure

Patient 5	
Neurological	No eye opening. No verbal response. No motor response
Cardiovascular	MAP above 65 on norepinephrine 0.5 µg/kg/min
Respiratory	Intubated. FiO2=100%. paO2=75 mmHg
Renal	On dialysis
Gastrointestinal	Bilirubin: 5 mg/dl
Hematology	Platelets: 30/nl

**Table 9:** Patient 6 MAP: mean arterial pressure

Patient 6	
Neurological	Eye opening to speech. Confused conversation. Localizes pain
Cardiovascular	MAP above 65 on norepinephrine 0.15 µg/kg/min
Respiratory	Intubated. FiO2=40%. paO2=120 mmHg
Renal	Creatinine: 2.0 mg/dl
Gastrointestinal	Bilirubin: 1.5 mg/dl
Hematology	Platelets: 130/nl

**Table 10:** Patient 7 MAP: mean arterial pressure

Patient 7	
Neurological	Eye opening to speech. Confused conversation. Localizes pain
Cardiovascular	MAP above 65 on norepinephrine 0.05 µg/kg/min
Respiratory	Ventilated. FiO2=30%. paO2=120 mmHg
Renal	Creatinine: 2.0 mg/dl
Gastrointestinal	Bilirubin: 1.0 mg/dl
Hematology	Platelets: 250/nl

**Table 11:** Patient 8 MAP: mean arterial pressure

Patient 8	
Neurological	Eye opening to speech. Confused conversation. Localizes pain
Cardiovascular	MAP above 65 on norepinephrine 0.05 µg/kg/min
Respiratory	Spontaneous breathing. FiO2=30%. paO2=120 mmHg
Renal	Creatinine: 2.5 mg/dl
Gastrointestinal	Bilirubin: 1.0 mg/dl
Hematology	Platelets: 250/nl

**Table 12:** Patient 9 MAP: mean arterial pressure

Patient 9	
Neurological	Eye opening to speech. No verbal response. Flexion to pain
Cardiovascular	MAP above 65 on norepinephrine 0.4 µg/kg/min
Respiratory	Spontaneous breathing. FiO2=50%. paO2=90 mmHg
Renal	Creatinine: 2.5 mg/dl
Gastrointestinal	Bilirubin: 4.0 mg/dl
Hematology	Platelets: 35/nl

**Table 13:** Patient 10 MAP: mean arterial pressure

Patient 10	
Neurological	Eye opening spontaneously. Oriented. Obeys command
Cardiovascular	MAP above 65 on norepinephrine 0.05 µg/kg/min
Respiratory	Spontaneous breathing. FiO2=30%. paO2=90 mmHg
Renal	Creatinine: 1.2 mg/dl
Gastrointestinal	Bilirubin: 1.0 mg/dl
Hematology	Platelets: 280/nl
